# New Insights into the Physiology of the Propionate Producers *Anaerotignum propionicum* and *Anaerotignum neopropionicum* (Formerly *Clostridium propionicum* and *Clostridium neopropionicum*)

**DOI:** 10.3390/microorganisms11030685

**Published:** 2023-03-07

**Authors:** Tina Baur, Peter Dürre

**Affiliations:** Institut für Mikrobiologie und Biotechnologie, Universität Ulm, Albert-Einstein-Allee 11, 89081 Ulm, Germany

**Keywords:** *Anaerotignum propionicum*, *Anaerotignum neopropionicum*, cell envelope, growth parameters, lignocellulosic hydrolysates, ethanol oxidation, propionate production

## Abstract

Propionate is an important platform chemical that is available through petrochemical synthesis. Bacterial propionate formation is considered an alternative, as bacteria can convert waste substrates into valuable products. In this regard, research primarily focused on propionibacteria due to high propionate titers achieved from different substrates. Whether other bacteria could also be attractive producers is unclear, mostly because little is known about these strains. Therefore, two thus far less researched strains, *Anaerotignum propionicum* and *Anaerotignum neopropionicum*, were investigated with regard to their morphologic and metabolic features. Microscopic analyses revealed a negative Gram reaction despite a Gram-positive cell wall as well as surface layers for both strains. Furthermore, growth, product profiles, and the potential for propionate formation from sustainable substrates, i.e., ethanol or lignocellulosic sugars, were assessed. Results showed that both strains can oxidize ethanol to different extents. While *A. propionicum* only partially used ethanol, *A. neopropionicum* converted 28.3 mM ethanol to 16.4 mM propionate. Additionally, the ability of *A. neopropionicum* to produce propionate from lignocellulose-derived substrates was analyzed, leading to propionate concentrations of up to 14.5 mM. Overall, this work provides new insights into the physiology of the *Anaerotignum* strains, which can be used to develop effective propionate producer strains.

## 1. Introduction

Propionate production is native to various members of many bacterial genera including, e.g., *Propionibacterium*, *Clostridium*, *Veillonella*, *Roseburia*, *Megasphaera*, and *Bacteroides* [1]. This list can be extended when including bacteria that are usually non-propionigenic but can produce this acid with certain substrates or under specific growth conditions such as *Escherichia coli* when grown with *L*-threonine under anaerobic conditions [2], *Acetobacterium woodii* and *Eubacterium maltosivorans* with 1,2-propanediol as a substrate [3,4], and *Clostridium kluyveri* when provided with propanol instead of ethanol [5]. Of all propionate producers, propionibacteria have received most attention, probably because they have been known for almost 145 years. The first mention of bacterial propionate production was in 1878 by Albert Fitz, who, although not referring to the bacteria as propionibacteria, discovered the basic stoichiometry of propionic acid fermentation from lactate [6]. Further studies including the isolation and description of numerous *Propionibacterium* species followed in the early 20th century by several authors [7,8,9]. Afterwards, propionibacteria have been widely studied with regards to their capability to produce high amounts of propionate via the Wood–Werkman cycle [1,10]. Due to the unique metabolism of propionibacteria, their substrate-to-propionate turnover is quite effective compared to other propionigenic bacteria, thus making them attractive for commercial propionate production [1,10]. The long history and the efficient metabolism of propionibacteria might be reasons why other native propionate producers have been neglected when it comes to investigating their general properties and growth kinetics, as well as assessing their potential as commercial and, nowadays more important, sustainable propionate producers. Two examples are *Anaerotignum propionicum* and *Anaerotignum neopropionicum*, formerly known as *Clostridium propionicum* and *Clostridium neopropionicum*, which have not been studied in depth after their original isolation in 1946 and 1982, respectively [11,12]. Only recently, *Anaerotignum neopropionicum* has been studied in greater detail as it was successfully used in a synthetic co-culture with *Acetobacterium wieringae* for propionate production from carbon monoxide [13] as well as for the development of a genome-scale metabolic model with its typical acrylate pathway [14]. These recent works have shed some light on the potential of *A. neopropionicum* to be used as a propionate producer strain. However, most of the available publications, excluding the original description of *A. neopropionicum* [15] and the most recent work [14], just studied the growth kinetics or some properties of the strain. Even less is known about *A. propionicum*, which previously has only been studied with regard to the activities of its acrylate pathway enzymes [16,17,18,19,20,21], been used as a reference strain for close relatives, e.g., other *Anaerotignum* strains [22,23,24], or been found in reactor microbiomes, enrichment cultures, or complex microbial communities such as in the human gut [25,26,27]. Therefore, the present study dealt with the characterization of both *Anaerotignum* strains on a morphologic and metabolic level to expand the knowledge about the physiology of the strains. We opted to determine the structure of the cell envelopes as well as assess the growth and product profiles of both strains when cultivated with different carbon sources to gather insights into the metabolic processes underlying substrate consumption and product formation. Furthermore, the utilization of ethanol and lignocellulosic sugars was analyzed to draw a conclusion as to whether the strains would be suitable as sustainable propionate producer strains. For these purposes, the strains were investigated using electron microscopy and growth, and product formation was analyzed using gas chromatography and high-performance liquid chromatography. Among other findings, this is the first study to present growth data for *A. propionicum* including its product profile with different substrates and the observation that *A. neopropionicum* can grow with complex lignocellulose-based substrates, which qualifies this strain as a sustainable propionate producer.

## 2. Materials and Methods

### 2.1. Bacterial Strains and Cultivation

The bacterial strains used in this study are listed in Table 1. *Anaerotignum* strains were obtained from DSMZ (Deutsche Sammlung von Mikroorganismen und Zellkulturen, Brunswick, Germany) and cultivated under strictly anaerobic conditions. *A. propionicum* was grown at 37 °C in DSMZ medium 156, which contained (per liter) 3 g peptone, 4 g yeast extract, 0.1 g MgSO_4_ × 7 H_2_O, 18 mg FeSO_4_ × 7 H_2_O, 5 mL potassium phosphate buffer (1 M, pH 7.1), 2.5 mL saturated CaSO_4_ solution, 1 g NaHCO_3_, 0.3 g *L*-cysteine-HCl × H_2_O, and 1 mg resazurin. The initial pH was set to 7.2 using NaOH. Cultivation of *A. neopropionicum* was performed at 30 °C using DSMZ medium 318b, which contained (per liter) 0.3 g KH_2_PO_4_, 0.6 g NaCl, 0.1 g MgCl_2_ × 6 H_2_O, 80 mg CaCl_2_ × 2 H_2_O, 10 mL Trace element solution ([28] mod., for details see Table S1), 10 mL Vitamin solution [29], 0.5 g yeast extract, 0.5 g peptone, 4 g KHCO_3_, 1 g NH_4_Cl, 0.3 g *L*-cysteine-HCl × H_2_O, 0.3 g Na_2_S × 9 H_2_O, and 1 mg resazurin. The pH was set to 7.2 using HCl before autoclaving.

Cultivation was carried out in 50 mL of the respective medium in 125-mL Müller–Krempel bottles (Müller & Krempel AG, Bülach, Switzerland) with a N_2_:CO_2_ headspace (80:20%) and supplementation of respective carbon sources unless otherwise indicated. Substrates were added after autoclaving from sterile, anaerobic stock solutions. Excello^TM^ stock solutions were prepared by firstly diluting respective pure suspensions obtained from Borregaard (Sarpsborg, Norway) to a concentration of 40% (*v*/*v*) and then filter-steri-lized in an anaerobic cabinet. For growth experiments, diluted Excello^TM^ solutions were supplemented to a final concentration of 2% (*v*/*v*). Pre-cultures of both strains were set up using cells grown in 5 mL of the respective medium and used to inoculate the main cultures. *B. subtilis* and *E. coli* XL1-Blue MRF’ served as reference strains for Gram staining and were cultivated aerobically in Luria–Bertani medium [30] under shaking conditions (180 rpm) at 30 and 37 °C, respectively.

### 2.2. Analytical Methods

Bacterial growth was monitored by measurement of the optical density at a wavelength of 600 nm (OD_600_) using an “Ultrospec^TM^ 3100 pro UV/Visible” spectrophotometer (Amersham Biosciences Europe GmbH, Freiburg, Germany).

The metabolic products acetate, propionate, butyrate, isobutyrate, isovalerate, ethanol, and propanol were quantified using gas chromatography (GC). For that purpose, 2-mL culture samples were withdrawn during growth experiments and centrifuged (15,000× *g*, 30 min, 4 °C). Subsequently, 480 µL of supernatant were filled into 2-mL crimp vials (CS-Chromatographie Service GmbH, Langerwehe, Germany), acidified by addition of 20 µL 2 M HCl, and sealed with aluminum caps. GC analysis of prepared samples was performed as previously described [31] with a few adjustments. In brief, 1 µL of prepared sample was injected (225 °C) into a “Clarus 600” gas chromatograph (PerkinElmer, Inc., Waltham, MA, USA) equipped with an “Elite-FFAP” capillary column (inner diameter 0.32 mm × 30 m, PerkinElmer, Inc., Waltham, MA, USA) and a flame ionization detector (300 °C), operating with H_2_ (45 mL min^−1^) and synthetic air (79.5% N_2_ + 20.5% O_2_ at 450 mL min^−1^) as detector gases. Samples were analyzed using a 3-step temperature profile (80 °C for 2 min followed by an increase in temperature to 250 °C with a rate of 30 °C min^−1^, finally 250 °C were held constant for 1 min). H_2_ served as carrier gas (2.25 mL min^−1^ flow rate). Defined external standards containing all substances were prepared for calibration purposes.

Quantification of sugars (glucose, xylose, Excello^TM^-95/99), alanine, and lactate was achieved using high-performance liquid chromatography (HPLC). All HPLC samples were prepared as previously described for GC; however, no acidification was performed. Detection of glucose, xylose, and lactate was achieved using an “Infinity 1260” HPLC system (Agilent Technologies, Santa Clara, CA, USA) equipped with a “CS–Organic-Acid Resin” column (150 × 8 mm, CS-Chromatographie Service GmbH, Langerwehe, Germany; column temperature at 40 °C), a diode array detector (for lactate; operating at 210 nm), and a refraction index detector (RID; for glucose and xylose; operating at 35 °C). H_2_SO_4_ (5 mM) was used as mobile phase at a flow rate of 0.7 mL min^−1^. Determination of sugar concentrations in Excello^TM^ substrates was performed using an “ELITE LaChrom” HPLC system (Hitachi High Technologies America, Inc., Schaumburg, IL, USA) with an integrated “NUCLEOGEL SUGAR Pb” column (300 × 7.8 mm, Macherey-Nagel GmbH & Co. KG, Düren, Germany) and an RID (operating at 45 °C). Separation was achieved at 80 °C using water as mobile phase (flow rate 0.4 mL min^−1^). Again, all compounds were quantified using defined external standards. Alanine consumption by *A. propionicum* was determined using the HPLC method described by [32] with a few alterations. Instead of an HP instrument, HPLC was carried out using a “1260 Infinity II” system (Agilent Technologies, Santa Clara, CA, USA) including a “multohyp octyldecyl silane” column (particle size 5 µm, 125 × 4 mm, CS-Chromatographie Service GmbH, Langerwehe, Germany) and a fluorescence detector (excitation 230 nm, emission 450 nm). Quantification of alanine was achieved at 40 °C using 100 mM sodium acetate and methanol (0.8 mL min^−1^) as mobile phase after automatic pre-column derivatization with *ortho*-phthaldialdehyde. *L*-ornithine served as an internal standard (at 100 µM) and was used in an 8-point calibration curve for calculation of alanine concentrations.

### 2.3. Gram Staining and Microscopy

Gram staining was performed as previously described [33] using *B. subtilis* DSM 402 and *E. coli* XL1-Blue MRF’ as reference strains for Gram-positive and Gram-negative staining, respectively. In brief, 10 µL of cell culture were air-dried and heat-fixed on an object slide. Subsequently, cells were covered with “Gram’s crystal violet solution” (Sigma-Aldrich GmbH, Steinheim, Germany) for 3 min. Thereafter, cells were washed with “Lugol’s iodine solution” (Carl Roth GmbH & Co. KG, Karlsruhe, Germany), then covered with “Lugol’s iodine solution” for 1 min and washed with 96% (*v*/*v*) ethanol until complete discoloration. After removing residual ethanol with water, cells were stained with “Gram’s safranin solution” (Sigma-Aldrich GmbH, Steinheim, Germany) for 1 min. Finally, cells were washed with water. Stained cells were observed using a “Zeiss AXIO Observer Z1” microscope (Carl Zeiss AG, Oberkochen, Germany) at a 100-fold magnification. For investigation of cell surface and cell wall structure of *Anaerotignum* strains, cells were imaged using scanning and transmission electron microscopy. After cultivating the strains in 5 mL of respective medium to an app. OD_600_ of 0.5, cells were harvested at 2600× *g* for 10 min at room temperature. A total of 4 mL of supernatant was discarded, and cells were resuspended in the remaining liquid. Then, cells were prepared for electron microscopy at the “Zentrale Einrichtung Elektronenmikroskopie” (University of Ulm, Ulm, Germany) as previously described [34]. First, cells were fixed using fixation solution (0.2 M phosphate-buffered saline (PBS, pH 7.3), 5% (*v*/*v*) glutaraldehyde, 2% (*w*/*v*) sucrose) and then washed five times with 0.01 M PBS. Post-fixation was achieved using 2% (*v*/*v*) aqueous osmium tetroxide. Subsequently, cells were dehydrated using a graded series of propanol (final step in 100% propanol) and then block-stained in 1% (*w*/*v*) uranyl acetate. For scanning electron microscopy, cells were completely dehydrated via critical-point-drying and coated with platinum. Cells imaged by transmission electron microscopy were embedded in epoxy resin, cut in ultra-thin sections, and contrasted with 0.3% (*w*/*v*) lead citrate. Finally, strains were visualized using a “Hitachi S-5200 UltraHigh Resolution FE” scanning electron microscope (Hitachi, Ltd. Corp., Chiyoda, Japan) and the “Jeol 1400” transmission electron microscope (Jeol GmbH, Freising, Germany).

## 3. Results

### 3.1. Investigation of Cell Envelope and Cell Surface

**Figure 1:**
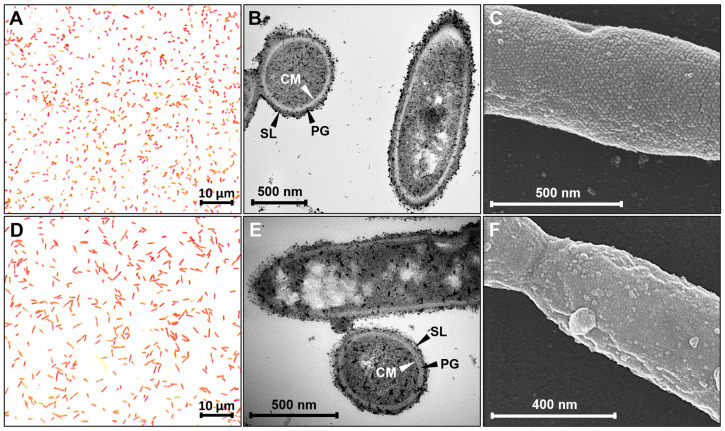
Overview of Gram type, cell wall structure, and surface layers of *A. propionicum* (**A**–**C**) and *A. neopropionicum* (**D**–**F**). Pictures were obtained using light microscopy, transmission, and scanning electron microscopy. (**A**,**D**) Results of Gram staining; (**B**,**E**) results of transmission electron microscopic analyses; (**C**,**F**) results of scanning electron microscopic analyses. CM, cytoplasmic membrane; PG, peptidoglycan; SL, surface layer.

The structure of the cell envelope of both *Anaerotignum* strains was assessed using different types of microscopy. A first result was obtained using Gram staining, which was negative for both strains when compared to the reference strains *B. subtilis* and *E. coli* (Figure 1A,D and Figure S1). A more detailed analysis of the cell envelopes was performed using scanning and transmission electron microscopy. Results revealed quite thick peptidoglycan layers in both strains (Figure 1B,E), which is typical for Gram-positive bacteria and thus stands in contrast to the negative staining results. The average width of the peptidoglycan was determined to be app. 25 to 27 nm. Transmission electron microscopy also showed a surrounding layer for both bacterial strains, which seems to loosely cover the cells and is less compact in structure than the well-organized peptidoglycan. Analysis of the cells via scanning electron microscopy showed that both strains are covered with a surface layer composed of protein subunits organized in a hexagonal or grid-like pattern (Figure 1C,F). These subunits have an estimated average diameter of 16 to 19 nm in case of *A. propionicum*, whereas the surface layer of *A. neopropionicum* consists of smaller protein subunits with an estimated average diameter of 4 to 7 nm per subunit. Aside from the cell envelope, microscopic analyses confirmed the rod shape, the cell size (1.1 × 0.8 µm for *A. propionicum*; 2.1 × 0.6 µm for *A. neopropionicum*), as well as the presence of filamentous structures for both strains (Figure 1 and Figure S2). While *A. propionicum* only produces single filaments, *A. neopropionicum* cells seem to produce many filaments that connect adjacent cells (Figure S2). Furthermore, white inclusions were observed in both bacteria as is visible in Figure 1.

### 3.2. Investigation of Metabolic Capabilities

**Figure 2:**
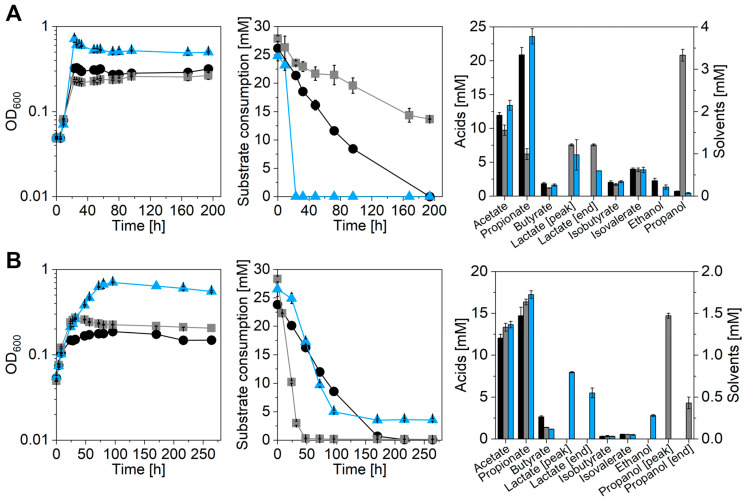
Overview of growth experiments conducted with *A. propionicum* and *A. neopropionicum* using different carbon sources. (**A**) Growth (OD_600_, (**left**)), substrate consumption (**middle**), and product formation (**right**) of *A. propionicum*; (**B**) growth (**left**), substrate consumption (**middle**), and product formation (**right**) of *A. neopropionicum*. Black circles, growth with *D*-lactate; grey squares, growth with ethanol; blue triangles, growth with *L*-alanine (*A. propionicum*) or xylose (*A. neopropio-nicum*). Error bars represent standard deviations, *n* = 3.

The characterization of the metabolic profile and the growth parameters was achieved by growing cells of *A. propionicum* and *A. neopropionicum* using different carbon sources. The emphasis was set on the utilization of substrates that already are or can potentially be produced from renewable resources or waste gases and are hence interesting for sustainable production of platform chemicals such as propionate. For this purpose, different growth experiments were conducted. In a first experimental set-up, ethanol oxidation by *Anaerotignum* strains was investigated. As references, both strains were cultivated in presence of *D*-lactate and a strain-specific carbon source, i.e., *L*-alanine for *A. propionicum* and xylose for *A. neopropionicum*. Results of the growth experiments are summarized in Figure 2 and Table 2.

Both strains showed growth in presence of all provided substrates, however, best growth was achieved with *L*-alanine and xylose as maximal OD_600_ and growth rate were the highest with these carbon sources (Table 2). Supplementation of *D*-lactate or ethanol only resulted in diminished growth with the max. OD_600_ ranging between 0.2 and 0.3 for both strains. *D*-lactate was fully depleted within app. 200 h by both bacterial strains, whereas consumption of the other substrates was different. While *A. neopropionicum* utilized all provided ethanol quite fast (depleted after 48 h), *A. propionicum* only slowly and only partially oxidized the ethanol leaving 13.7 mM untouched after 194 h of cultivation. In contrast to *D*-lactate and ethanol, the strain-specific carbon sources were either completely consumed (*L*-alanine by *A. propionicum*) or again only partially metabolized (3.5 mM xylose left by *A. neopropionicum*). Product distribution turned out to be similar for both strains and all substrates except for the ethanol-grown culture of *A. propionicum.* In this case, acetate and propionate titers were reduced to 7.6 and 9.7 mM, respectively, compared to all other cultures, which reached concentrations between 11.9 and 13.6 mM acetate and 14.7 and 23.6 mM propionate (Figure 2 and Table 2). Instead, *A. propionicum* produced proportionally more lactate (7.6 mM) when ethanol was supplemented, thus creating a 1 to 0.6 and to 0.8 ratio of acetate to propionate and lactate, respectively. In contrast, the other lactate-producing cultures, which were *A. propionicum* cultivated with *L*-alanine and *A. neopropionicum* cultivated with xylose, reached a ratio of roughly 1 to 1.5 and 0.5 and thus produced up to app. 3-times more propionate. Moreover, produced lactate was partially reassimilated by *Anaerotignum* strains when cultivated with *L*-alanine or xylose, whereas it was accumulated by *A. propionicum* when grown with ethanol. Other products detected were butyrate, ethanol, and propanol, as well as isobutyrate and isovalerate. Butyrate titers were rather low for both strains and with all substrates as the maximal concentrations ranged between 1.2 and 2.6 mM. Similarly low amounts were detected for isobutyrate and isovalerate, which were produced in all cultures independent of the substrate. The isobutyrate to isovalerate ratio was always found to be app. 1:2 for both *Anaerotignum* strains. Absolute titers achieved were 1.7–2.1 mM isobutyrate and 3.9–4.0 mM isovalerate in *A. propionicum* and 0.3 mM isobutyrate and 0.5–0.6 mM isovalerate in *A. neopropionicum* cultures. Another interesting finding was propanol production of the strains when cultivated with ethanol. While *A. propionicum* accumulated 3.3 mM propanol over the course of the whole cultivation span, *A. neopropionicum* built up 1.5 mM propanol during early exponential growth (until 32 h of cultivation) and afterwards reassimilated most of it, leaving 0.4 mM at the end of the experiment. Ethanol remained a minor product for all *Anaerotignum* cultures as only traces of up to 0.4 mM or no ethanol at all were detected.

**Figure 3:**
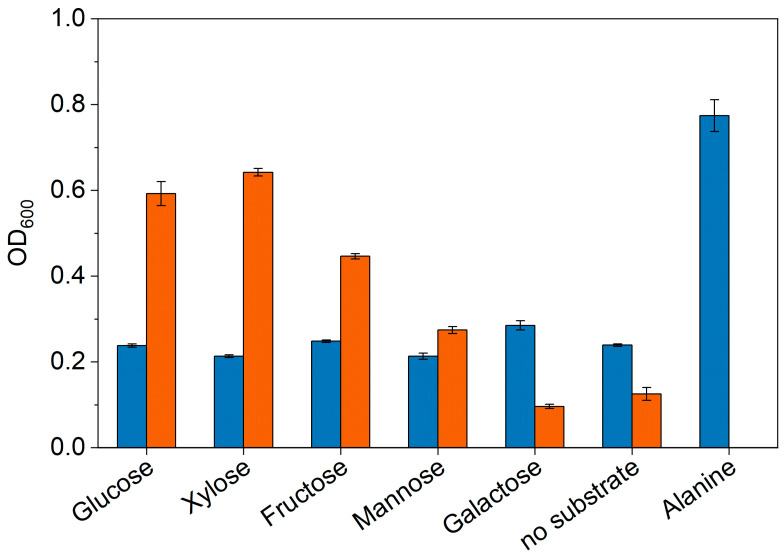
Growth of *A. propionicum* (blue) and *A. neopropionicum* (orange) in presence of 50 mM sugar. Cultures without supplementation of a substrate served as a negative control, addition of 50 mM *L*-alanine served as a positive control for growth of *A. propionicum*. Cultivation was performed in 5 mL respective medium with supplemented carbon sources. Starting OD_600_ was set to 0.1 for *A. propionicum* and 0.05 for *A. neopropionicum*. Error bars represent standard deviations, *n* = 3.

Aside from ethanol oxidation, sugar utilization of *Anaerotignum* strains was investigated in detail. First, growth of the strains in presence of different sugars was evaluated qualitatively as presented in Figure 3. In comparison to the negative controls harboring no additional substrate, only *A. neopropionicum* showed growth in presence of glucose, xylose, fructose, and mannose with a preference for glucose and xylose as OD_600_ was app. 5-fold higher than the negative control and 1.4-fold higher than the next best sugar (fructose). Growth was not supported by addition of galactose. In contrast, *A. propionicum* was only able to grow when *L*-alanine was supplemented. Addition of the different sugars had no positive effect on growth, as OD_600_ remained in the range of the negative control at a value of app. 0.25.

Since only *A. neopropionicum* turned out to be saccharolytic, its growth and the potential of this strain to produce propionate using the lignocellulosic hydrolysates Excello^TM^-95 and -99 (Borregaard, Sarpsborg, Norway; Table S2) was tested (Table 3). A culture grown with glucose served as reference. All cultures showed similar growth, reaching a maximal OD_600_ of 0.7 within 80 h and a growth rate of 0.04–0.05 h^−1^. The product profile was also rather similar, as the main products were acetate (13.5–15.4 mM) and propionate (13–14.5 mM) with most propionate produced using Excello^TM^-95. Minor side products were butyrate (1.5–1.7 mM), lactate (1.9–2.4 mM), ethanol (0.3–0.5 mM), isobutyrate (0.2–0.3 mM), and isovalerate (0.5 mM). Propanol was not detected.

Analysis of sugar consumption revealed that *A. neopropionicum* cultivated with Excello^TM^ substrates simultaneously utilized glucose and xylose, incompletely however (Figure S3). In total, *A. neopropionicum* metabolized 14.2 mM glucose and 3.6 mM xylose when grown with Excello^TM^-95, and 15 mM glucose as well as 5.5 mM xylose when Excello^TM^-99 was added (Table 3). Most of the supplied sugar was left over as 61.1 mM glucose and 4.7 mM xylose (Excello^TM^-95) as well as 48.2 mM glucose and 3.6 mM xylose (Excello^TM^-99) were detected at the end of the experiment (Figure S3). Aside from glucose and xylose, 6 mM galactose, 3 mM mannose, and 0.3 mM cellobiose were also detected in the Excello^TM^ mixtures, however, neither of these sugars was utilized by *A. neopropionicum*. The culture grown on pure glucose also did not fully deplete the provided substrate since only 16.8 mM of the provided 54.1 mM were consumed (Figure S3).

**Table 3:** Overview of growth parameters, substrate consumption, and product formation of *A. neopropionicum* when cultivated with different sugar substrates (1% (*w*/*v*) glucose or 2% (*w*/*v*) Excello^TM^). Product concentrations refer to the mean titers at the end of cultivation (*n* = 3). µ, growth rate; t_D_, doubling time; -, not detected.

	1% Glucose	2% Excello^TM^-95	2% Excello^TM^-99
**Growth parameters**			
max. OD_600_	0.7	0.7	0.7
µ [h^−1^]	0.05	0.04	0.05
t_D_ [h]	13.3	15.9	15.3
**Substrate consumption [mM]**			
Glucose	16.8	14.2	15.0
Xylose	-	3.6	5.5
**Product profile [mM]**			
Acetate	13.5	15.4	14.1
Propionate	13.0	14.5	13.4
Butyrate	1.5	1.7	1.5
Lactate	1.9	2.2	2.3
Ethanol	0.5	0.3	0.3
Propanol	-	-	-
Isobutyrate	0.3	0.2	0.2
Isovalerate	0.5	0.5	0.5

## 4. Discussion

According to the Gram reaction, the *Anaerotignum* strains should be classified as Gram-negative thereby confirming the findings of previous studies [11,15]. However, in contrast to the staining result, transmission electron micrographs clearly showed a Gram-positive-type cell wall structure as the peptidoglycan layer is quite thick in both strains. Since the Gram staining method relies on the thickness of the peptidoglycan layer, which, when thick enough, retains the Gram stain complex and leads to a blue-purple color, it seems as if this layer in the present cases is too thin and this leads to decolorization during wash steps. In fact, it was previously hypothesized that a minimum thickness is required to retain the Gram stain complex [35]. Typical Gram-positive cell walls comprise peptidoglycan layers with a thickness of 30 to 100 nm [36,37]. This would provide a reasonable explanation as to why *Anaerotignum* strains stained Gram-negative despite having a Gram-positive cell wall as transmission electron microscopic analyses revealed a thickness of app. 25 to 27 nm and thus slightly less than the assumed minimum of 30 nm. This misleading staining result is not uncommon as the peptidoglycan content of bacteria varies depending on the growth phase, the growth rate, as well as the growth conditions [38,39]. Apparently, *Clostridium* sp. frequently have rather thin peptidoglycan layers due to rapid growth and a disjointed cell wall turnover, leading to a negative Gram reaction [39,40]. This is backed up by multiple examples of Gram-variable strains, i.e., clostridia that stain Gram-negative but are in fact Gram-positive, e.g., *Clostridium phytofermentans*, *Clostridium polysaccharolyticum*, *Clostridium thermocellum*, *Clostridium thermopalmarium*, and *Clostridium xylanolyticum* [40,41,42,43,44]. According to [39], Gram-variable species, to which the two *Anaerotignum* strains belong, are more vulnerable due to the thinner peptidoglycan layer and therefore often have a surface layer as a protective coat, and to help strengthening the underlying cell wall. As can be seen in Figure 1, both *Anaerotignum* strains possess such a surface layer as do all other mentioned Gram-variable species [40,41,42,44,45] which supports this hypothesis well. In addition to the specificities of the cell envelope, transmission electron micrographs revealed electron lucent inclusions in both *Anaerotignum* strains (Figure 1). Such cytoplasmic electron transparent inclusions can frequently be observed in bacteria as they often deposit polysaccharides such as glycogen, starch, or amylopectin as energy reserves [46]. These storage compounds appear as white inclusions in the cytoplasm when investigated via transmission electron microscopy as previously shown for various bacteria such as *Clostridium pasteurianum*, *Clostridium polysaccharolyticum*, and *Megasphaera elsdenii* [42,46,47]. Both *Anaerotignum* strains possess genes encoding the glycogen synthesis pathway, however, other possible storage compounds are lipid droplets or polyamines [48]. Further observations regarding the cell shape and size as well as the presence of flagella confirm previous findings [11,15].

Investigation of growth and product profiles of both *Anaerotignum* strains revealed interesting metabolic features. Ethanol oxidation has previously been reported for both strains [15,22,23]; however, it has not been described that the two strains differ in their capability to do so. While *A. neopropionicum* consumed all ethanol within 48 h at a rate of 0.39 mM h^−1^, *A. propionicum* only partially oxidized it with a rate of 0.07 mM h^−1^ with 13.7 mM remaining at the end of cultivation. Since *A. neopropionicum* consumed all provided ethanol, high product concentrations were achieved (13.3 mM acetate and 16.4 mM propionate). In contrast, *A. propionicum* only produced 9.7 mM acetate and 6.2 mM propionate, which is far less than with any other substrate (Figure 2 and Table 2). However, proportionally more lactate was detected, indicating that *A. propionicum* seems to stop at the stage of lactate instead of going all the way to propionate. Increased propanol concentrations can be explained as a means to dispose of excess reducing equivalents that are formed during ethanol oxidation to acetyl–CoA. The process of ethanol oxidation is energetically challenging as the redox potential of ethanol/acetaldehyde (E_0_′ = −190 mV) is higher than the redox potential of NAD^+^ (E_0_′ = −320 mV), which acts as the cofactor in the conversions of ethanol to acetyl–CoA and thus poses an energetic barrier [49]. To overcome this barrier, the strains can use their respective bifunctional acetaldehyde/alcohol dehydrogenases (AdhE; CLNEO_13930; CPRO_10650; [50,51]) that couple both steps of ethanol oxidation (ethanol to acetaldehyde to acetyl–CoA) in one enzyme complex and this way reduce the energetic gap between ethanol/acetaldehyde and NAD^+^ as has previously been hypothesized for *A. woodii* [49]. Formed acetyl–CoA is then converted to acetate and propionate as previously described [14,33]. While acetate formation contains one energy-conserving step in the acetate kinase reaction, propionate formation under these conditions is an energy-consuming process. To get to propionate, one CO_2_ needs to be fixed during pyruvate formation, which is achieved by the pyruvate:ferredoxin oxidoreductase (Pfor). In the Pfor reaction, one reduced ferredoxin (Fd_red_) is oxidized per fixed CO_2_. To provide the necessary Fd_red_, the Rnf complex of the strains needs to operate in reverse direction, which is accompanied by the discharge of an ion gradient. This ion gradient in turn needs to be built up by the ATPase at the expense of ATP, thereby making ethanol oxidation not only a thermodynamically but also an energetically challenging process [14,33]. Since the AdhEs of *A. propionicum* and *A. neopropionicum* share a 92% sequence similarity on the protein level, it remains unclear as to why *A. neopropionicum* is much more effective in its ethanol metabolism than *A. propionicum*. It might be possible that this difference is a result of a different metabolic or regulatory set-up due to differently expressed *adhE* or other genes involved in ethanol metabolism, which could only be investigated via a transcriptomic or proteomic study.

A similar energetically challenging situation is faced when ethanol is substituted by *D*-lactate as a substrate. After its uptake, *D*-lactate is partially oxidized to acetate and partially reduced to propionate. The reduction to propionate requires reducing equivalents, which are generated during lactate oxidation to acetate, thereby closing the redox cycle. However, the oxidation of *D*-lactate with NAD^+^ as a cofactor is unfavorable as the redox potential of the *D*-lactate/pyruvate pair (E_0_′ = −190 mV) is far higher than the reduction potential of NAD^+^/NADH (E_0_′ = −320 mV) and thus would require the electrons to travel uphill [52,53]. Still, lactate oxidation is possible in these strains as *D*-lactate is completely exhausted (Figure 2). *A. woodii* manages lactate oxidation via an electron-confurcating lactate dehydrogenase complex in which Fd_red_ enables bridging of the gap between lactate and NAD^+^ [53]. In this process, the exergonic electron flow from Fd_red_ to NAD^+^ delivers the energy necessary to transfer electrons from lactate to NAD^+^. For this purpose, the lactate dehydrogenase forms a complex with two electron-transferring flavoproteins (ETFs), all of which are encoded in the *lct* operon [52,53]. Similar genetic arrangements can be found in many anaerobes including *Clostridium ljungdahlii*, *Clostridium saccharoperbutylacetonicum*, *Clostridium botulinum*, *Clostridium homopropionicum*, and *Eubacterium limosum* indicating a general mode of lactate oxidation in anaerobic bacteria [33,52,53,54]. However, no evidence of such an operon can be found in the genomes of *A. propionicum* and *A. neopropionicum*; only single *D*-lactate dehydrogenase genes without any surrounding ETF genes are present. This would mean that these strains have found an alternative way to manage lactate oxidation despite the energetic challenges this process presents. One possible explanation could be that the resulting pyruvate is immediately converted to acetyl–CoA by the Pfor thereby leading to the production of Fd_red_, which can contribute to NADH formation and energy conservation, and at the same time push the equilibrium of the lactate dehydrogenase reaction towards lactate oxidation. This strategy is also employed by mammalian cells as lactate oxidation with NAD^+^ is enabled by direct pyruvate removal at the mitochondrial membrane [52]. In addition, lactate uptake by the *Anaerotignum* strains requires a fair amount of cellular energy as it is achieved via a cotransport with H^+^ ions, thereby simultaneously discharging the H^+^ gradient across the membrane [33]. This gradient has to be built up by ATPases at the expense of ATP and thus is as costly as the previously discussed ethanol oxidation. In total, growth on low-energy substrates such as ethanol and *D*-lactate requires a high energy input from the strains, which explains the lowered max. OD_600_ (0.2–0.3) compared to the other high-energy substrates, i.e., sugars or *L*-alanine (OD_max_ 0.7). When *Anaerotignum* strains were cultivated on said high-energy substrates (*L*-alanine, xylose, or glucose), max. OD_600_ as well as acetate and propionate concentrations were the highest compared to all other substrates tested (Figure 2; Table 2 and Table 3). This is due to the fact that the overall ATP expense during utilization of the substrates is lower, because it is compensated during glycolysis and/or enough reducing equivalents are generated during conversion of the substrates, thereby preventing a reversal of the ATPase and Rnf reactions and increasing the available amount of ATP for growth.

**Figure 4:**
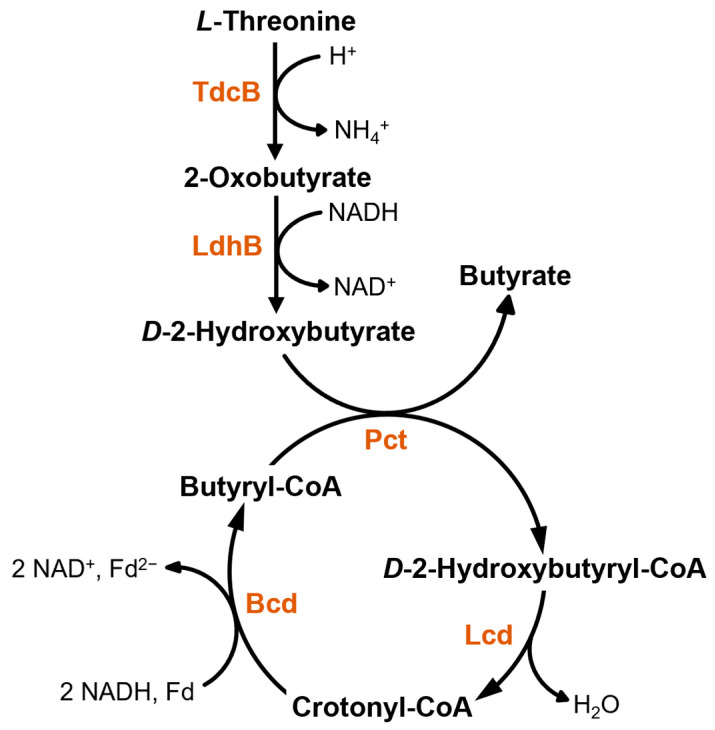
Proposed pathway for butyrate formation in *Anaerotignum* strains (based on [19]). TdcB, *L*-threonine dehydratase (CLNEO_20890, CPRO_03900); LdhB, *L*-lactate dehydrogenase (CLNEO_01790, CPRO_00680); Pct, propionate CoA-transferase (CLNEO_17700, CPRO_08400); Lcd, lactoyl–CoA dehydratase (CLNEO_17710-17730, CPRO_08370-08390); Bcd, butyryl–CoA dehydrogenase (CLNEO_29840-29850 or CLNEO_21740-21760, CPRO_28160-28180).

Both strains produced small amounts of butyrate (1.2–2.6 mM) with all tested substrates as has previously been observed by multiple groups [13,14,15,23]. Typically, butyrate formation is achieved via chain elongation of acetyl–CoA to butyryl–CoA, which can then be turned over to butyrate by phosphotransbutyrylase and butyrate kinase, as is the case for solventogenic clostridia [55], or alternatively by a CoA-transferase as described for *C. kluyveri* [5]. In all cases, bacteria encode respective enzymes in the *bcs* operon (butyryl–CoA synthesis operon) comprising the genes *crt*–*bcd*–*etfB*–*etfA*–*hbd* (coding for crotonase, butyryl–CoA dehydrogenase with its ETFs A and B, and 3-hydroxybutyryl–CoA dehydrogenase, respectively; [55]) as well as a thiolase gene (*thl*). A search for these genes in the genomes of the *Anaerotignum* strains did not provide positive results as neither strain harbors the full *bcs* operon or a thiolase-encoding gene. Only analogues of *bcd* and *etf* genes were identified in both genomes indicating the presence of a butyryl–CoA dehydrogenase. In addition, *A. propionicum* possesses a crotonase-encoding gene, which is not present in *A. neopropionicum*. Hence, it seems not to be possible for the *Anaerotignum* strains to produce butyrate via the “classical clostridial way”. Still, the authors of a recent study postulate butyrate formation in *A. neopropionicum* to happen via the typical route involving all mentioned enzymes [14]. While they could also find the butyryl–CoA dehydrogenase-encoding genes and none of the other genes (*crt*, *hbd*, *thl*), they claim to have found candidate enzymes to take over Crt and Hbd reactions. However, no thiolase or any other enzyme to catalyze the condensation of two acetyl–CoA molecules to acetoacetyl–CoA, the first step towards C_4_ compounds, was found. Since no thiolase seems to be present, which this and the previous study agree on, detected butyrate cannot be derived from acetyl–CoA. The same applies to *A. propionicum*, which also lacks a thiolase. Therefore, butyrate formation must happen in a different manner. The only other possible way is via *L*-threonine degradation as shown in Figure 4 and firstly described for *A. propionicum* [19]. In this pathway, *L*-threonine is first dehydrated to 2-oxobutyrate by a *L*-threonine dehydratase, which is subsequently reduced to *D*-2-hydroxybutyrate by a *L*-lactate dehydrogenase. By action of the propionate CoA-transferase, which is usually involved in propionate formation via the acrylate pathway [21], a CoA-moiety is transferred from butyryl–CoA to *D*-2-hydroxybutyrate thereby releasing butyrate. Butyryl–CoA can be formed from *D*-2-hydroxybutyrl–CoA by the action of the lactoyl–CoA dehydratase, which also is an acrylate pathway enzyme, and the identified butyryl–CoA dehydrogenase. Since both *Anaerotignum* media contain yeast extract and peptone, *L*-threonine most likely stems from these complex constituents. This is supported by the fact that growth experiments conducted with both strains and glycerol as the main substrate also yielded small amounts of butyrate (0.9 mM for *A. neopropionicum* and 1.2 mM for *A. propionicum*) despite glycerol not being utilized by either strain. As can be seen from Figure 4, the newly postulated butyrate formation pathway does not include an ATP-conserving step, which means that butyrate production is not directly linked to energy conservation. However, crotonyl–CoA reduction to butyryl–CoA leads to the formation of Fd_red_, which can possibly be turned over by the Rnf complex and thus contribute to energy conservation indirectly. Furthermore, NADH is oxidized in two reactions of the pathway indicating that butyrate formation in the *Anaerotignum* strains could be a way to dispose of excess reducing equivalents resulting from the oxidation of the substrates in the main pathways and this way generate Fd_red_ to increase the ATP gain.

Despite the close genetic relationship between the two *Anaerotignum* strains, there are metabolic differences between the two. Aside from the difference in the potential to oxidize ethanol and produce propionate from it, sugar utilization is very different. While *A. propionicum* turned out to be completely asaccharolytic, *A. neopropionicum* showed growth in the presence of glucose, xylose, fructose, and mannose (Figure 3). This is mostly in agreement with previous observations [11,15]; however, fructose and mannose utilization are new findings. Both strains possess the full genetic equipment for sugar metabolism, i.e., glycolytic, gluconeogenetic, and sugar transport systems, but only *A. neopropionicum* seems to be able to use them when cultivated in presence of sugars. It can only be speculated that this is the result of a different genetic expression pattern or of different regulatory networks that enable *A. neopropionicum* to metabolize various sugars and prevent *A. propionicum* from doing the same. The reasons for the asaccharolytic behavior of *A. propionicum* thus remain a mystery since sugars are probably the most favorable substrates for bacterial growth. *A. neopropionicum* was also able to grow with two different sugar mixtures derived from lignocellulose (Excello^TM^-95 and -99, composition presented in Table S2). Growth and product patterns were nearly identical to cultures grown with pure glucose (Table 3), which is probably due to the fact that both sugar mixtures mainly contain glucose. Xylose was used simultaneously but neither sugar was completely depleted (Figure S3). Other sugars detected in the mixtures, i.e., cellobiose, mannose, and galactose were not utilized, indicating that there might be carbon catabolite repression, at least in the case of cellobiose and mannose since galactose was also not utilized when provided as a single carbon source (Figure 3). Most propionate (14.5 mM) was produced with Excello^TM^-95, which highlights the potential of *A. neopropionicum* to produce this platform chemical from sustainable resources.

## 5. Conclusions

This study expanded the existing knowledge about the physiology of the two *Anaero-tignum* strains and provided new insights. This includes the classification of the strains as Gram-variable species with a surface layer surrounding the cells. Furthermore, this is the first study to provide basic growth and product profiles for *A. propionicum* as well as *A. neopropionicum* when cultivated with different substrates. A new pathway for butyrate formation was postulated, as the genetic equipment of the strains is not sufficient to produce butyrate via the classical chain elongation. Both strains are capable of ethanol oxidation; however, *A. neopropionicum* is much more effective in that regard. This strain also can produce propionate from lignocellulose-derived sugars making it an interesting and promising candidate for the sustainable production of propionate. Considering that ethanol can already be produced from industrial waste gas at commercial scale using *Clostridium autoethanogenum* [56,57], not only lignocellulose but also ethanol can be used as a sustainable substrate for *A. neopropionicum*, thus expanding the use of this strain for a green and possibly carbon-negative propionate production even further.

## Figures and Tables

**Table 1:** Bacterial strains used in this study.

Bacterial Strain	Relevant Characteristics	Origin
*Anaerotignum neopropionicum* DSM 3847	Type strain	DSMZ * GmbH, Brunswick, Germany
*Anaerotignum propionicum* DSM 1682	Type strain	DSMZ * GmbH, Brunswick, Germany
*Bacillus subtilis* DSM 402	*trp*^−^ or *ind*^−^	DSMZ * GmbH, Brunswick, Germany
*Escherichia coli* XL1-Blue MRF’	Δ(*mcrA*)183 Δ(*mcrCB*-*hsdSMR*-*mrr*)173 *endA1 supE44 thi*-1 *recA1 gyrA96 relA1 lac* (*F’proAB lacI^q^* ZΔM15 Tn10 (Tet^R^))	Agilent Technologies, Santa Clara, CA, USA

* German Collection of Microorganisms and Cell Cultures (DSMZ).

**Table 2:** Overview of growth parameters and product profiles of *A. propionicum* and *A. neopropionicum* when cultivated with different carbon sources. Product concentrations refer to the mean titers at the end of cultivation (*n* = 3). µ, growth rate; t_D_, doubling time; -, product not detected.

	*A. propionicum*			*A. neopropionicum*		
	Ethanol	*D*-Lactate	*L*-Alanine	Ethanol	*D*-Lactate	Xylose
**Growth parameters**						
max. OD_600_	0.3	0.3	0.7	0.3	0.2	0.7
µ [h^−1^]	0.09	0.11	0.15	0.05	0.04	0.05
t_D_ [h]	8.0	6.6	4.7	13.1	16.3	14.2
**Product profile [mM]**						
Acetate	9.7	11.9	13.4	13.3	12.0	13.6
Propionate	6.2	20.9	23.6	16.4	14.7	17.2
Butyrate	1.2	1.8	1.6	1.4	2.6	1.2
Lactate	7.6	-	3.7	-	-	5.5
Ethanol	-	0.4	0.2	-	-	0.3
Propanol	3.3	0.1	0.1	0.4	-	-
Isobutyrate	1.7	2.0	2.1	0.3	0.3	0.3
Isovalerate	3.9	4.0	3.9	0.5	0.6	0.5

## Data Availability

The data presented in this study are available on request from the corresponding author.

## Supplementary Materials

The following supporting information can be downloaded at: https://www.mdpi.com/article/10.3390/microorganisms11030685/s1, Table S1: Composition of trace element solution for DSMZ medium 318b; Table S2: Composition of Excello^TM^-95 and -99; Figure S1: Gram staining of *Anaerotignum* strains in comparison to *B. subtilis* and *E. coli*; Figure S2: Scanning electron micrographs of *A. propionicum* and *A. neopropionicum*; Figure S3: Sugar consumption of *A. neopropionicum* when cultivated with glucose or the lignocellulosic hydrolysates Excello^TM^-95 or Excello^TM^-99.
