# Supplementary material for: New Insights into the Physiology of the Propionate Producers Anaerotignum propionicum and Anaerotignum neopropionicum (Formerly Clostridium propionicum and Clostridium neopropionicum)
Source: Microorganisms. 2023 Mar 7;11(3):685. doi: 10.3390/microorganisms11030685 (PMC10053330; doi:10.3390/microorganisms11030685)
Supplement: Supplementary file 1 [file microorganisms-11-00685-s001.zip › microorganisms-2229040-supplementary.pdf]

Supplementary material

Microorganisms

Special Issue “Physiology, Genetic and Industrial Applications of Clostridia”

New insights into the physiology of the propionate producers *Anaerotignum propionicum* and *Anaerotignum neopropionicum* (formerly *Clostridium propionicum and Clostridium neopropionicum*)

Authors

Tina Baur[*] and Peter Dürre

Institut für Mikrobiologie und Biotechnologie, Universität Ulm, Albert-Einstein-Allee 11, 89081 Ulm, Germany

[*]Correspondence: Tina Baur

e-mail address: tina.baur@uni-ulm.de

telephone: (0049)731-50-22713

fax number: (0049)731-50-22719

Table S1. Trace element solution for DSMZ medium 318b ([28] mod.).

Compound	Amount
Nitrilotriacetic acid	12.8 g
$FeCl_2$ x 4 H_2O	1.0 g
$MnCl_2$ x 4 H_2O	0.1 g
$CoCl_2$ x 6 H_2O	30.0 mg
$CaCl_2$ x 2 H_2O	0.1 g
$ZnCl_2$	0.1 g
$CuCl_2$	20.0 mg
H_3BO_3	10.0 mg
Na_2MoO_4 x 2 H_2O	30.0 mg
$NiCl_2$ x 6 H_2O	0.1 g
NaCl	1.0 g
Na_2SeO_3 x 5 H_2O	30.0 mg
Na_2WO_4 x 2 H_2O	40.0 mg
H_2O	ad 1.0 l

Trace element solution was prepared by firstly dissolving nitrilotriacetic acid in water while adjusting pH to 6.5 with KOH. Then, all other substances were dissolved, and pH was adjusted to 6.5 using KOH.

Table S2. Composition of Excello™-95 and Excello™-99 according to Borregaard (Sarpsborg, Norway).

Compound	Excello™-95 [%]	Excello™-99 [%]
Glucose	78.5	73.2
Xylose	5.6	6.9
Mannose	6.3	8.7
Galactose	1.4	1.6
Arabinose	0.8	0.8
Fructose	1.7	1.5
Cellobiose	1.7	2.4
Gentiobiose	3.1	3.9
Lactic acid	0.1	0.2
Formic acid	0.1	0.1
Acetic acid	0.2	0.2
Glycerol	0.6	0.1

For preparation of anaerobic stock solutions, original pure suspensions obtained from Borregaard (Sarpsborg, Norway) were diluted to 40 % (*v/v*) and then filter-sterilized in an anaerobic cabinet. In the growth experiments, diluted stock solutions were used to supplement a total of 2 % (*v/v*) of the Excello™ mixtures.

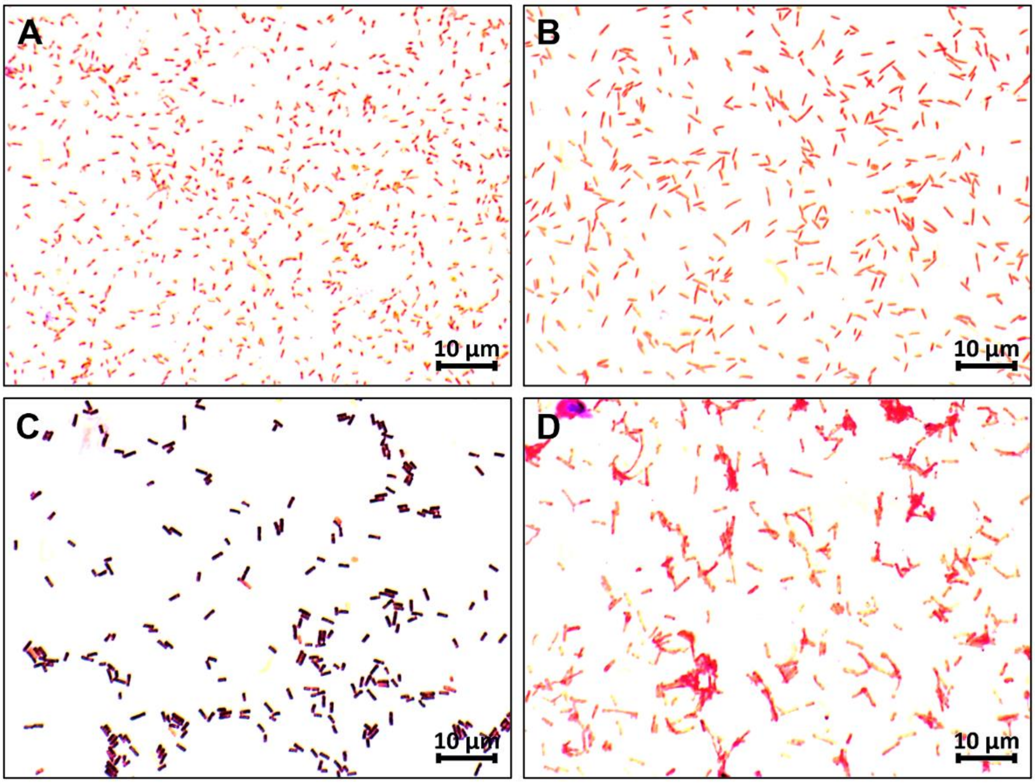

Figure S1. Gram staining of *A. propionicum* (A) and *A. neopropionicum* (B) in comparison to Gram-positive *B. subtilis* (C) and Gram-negative *E. coli* (D).

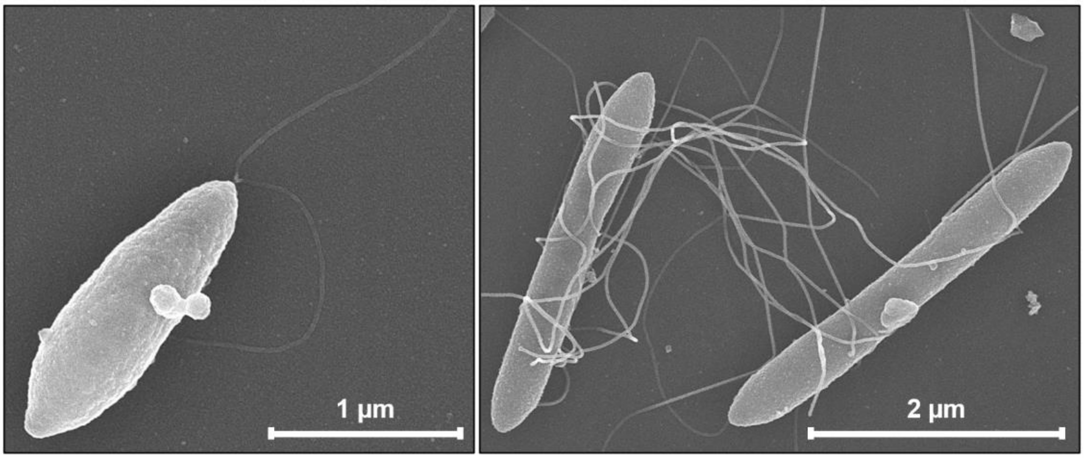

Figure S2. Scanning electron micrographs of *A. propionicum* (left) and *A. neopropionicum* (right) for visualization of filamentous structures.

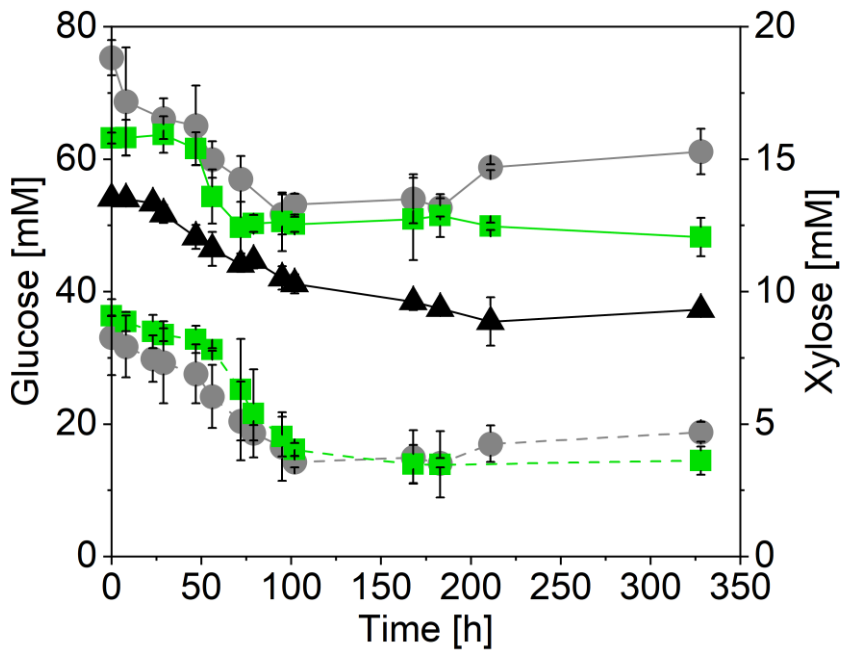

Figure S3. Sugar consumption of *A. neopropionicum* cultivated with glucose (black triangles) or lignocellulosic hydrolysates Excello^{TM}-95 (grey circles) or Excello^{TM}-99 (green squares). Solid lines, glucose consumption; dashed lines, xylose consumption. Cellobiose, mannose, and galactose consumption was omitted from the graph since no consumption was observed. Error bars represent standard deviations, *n* = 3.
